# Supplementary material for: Molecular Characterization of miRNAs in Myzus persicae Carrying Brassica Yellows Virus
Source: Biology (Basel). 2024 Nov 18;13(11):941. doi: 10.3390/biology13110941 (PMC11591976; doi:10.3390/biology13110941)
Supplement: Supplementary file 1 [file biology-13-00941-s001.zip › Supplementary Information.pdf]

Supplementary Information

Figure S1

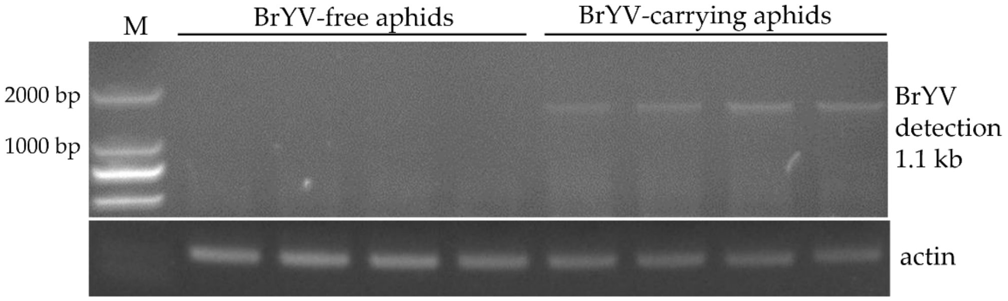

(a)

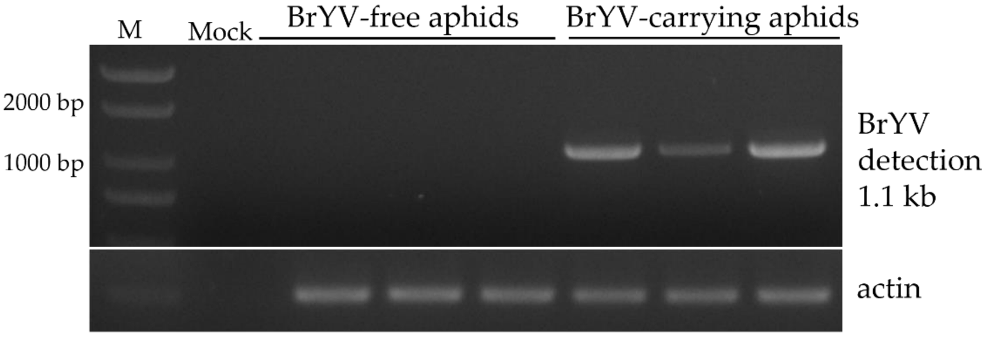

(b)

Figure S1. Confirmation of virus presence in BrYV-free and BrYV-carrying aphids using Reverse transcription-polymerase chain reaction (RT-PCR). (a) RT-PCR detection for treatment 1. **(b)** RT-PCR detection for treatment 2. M: DNA ladder. The specific primers BrC 2674F/ BrC3778R were used to detect the presence of BrYV. The quality of aphid samples was verified by amplifying actin (accession:XM_022309797) using primers actin F and actin R.

Figure S2

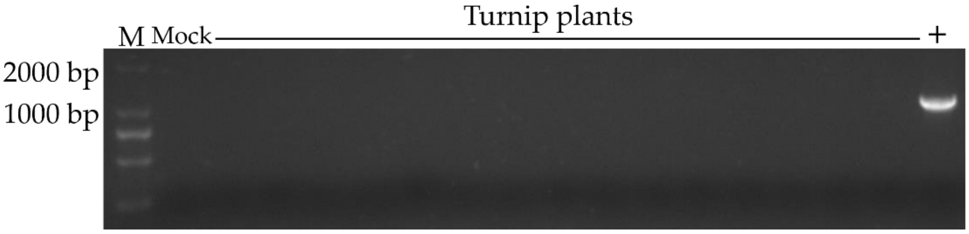

Figure S2. Turnip is verified as a nonhost of BrYV-C. Six aphids fed on the transgenic *A. thaliana* line 412 were transferred to turnip plants for 3 days, then aphids were eliminated using an insecticide. At 14 days after inoculation, systemic leaves were detected by RT-PCR. The result showed viruliferous aphids could not successfully transmit BrYV to turnip plants.

Figure S3

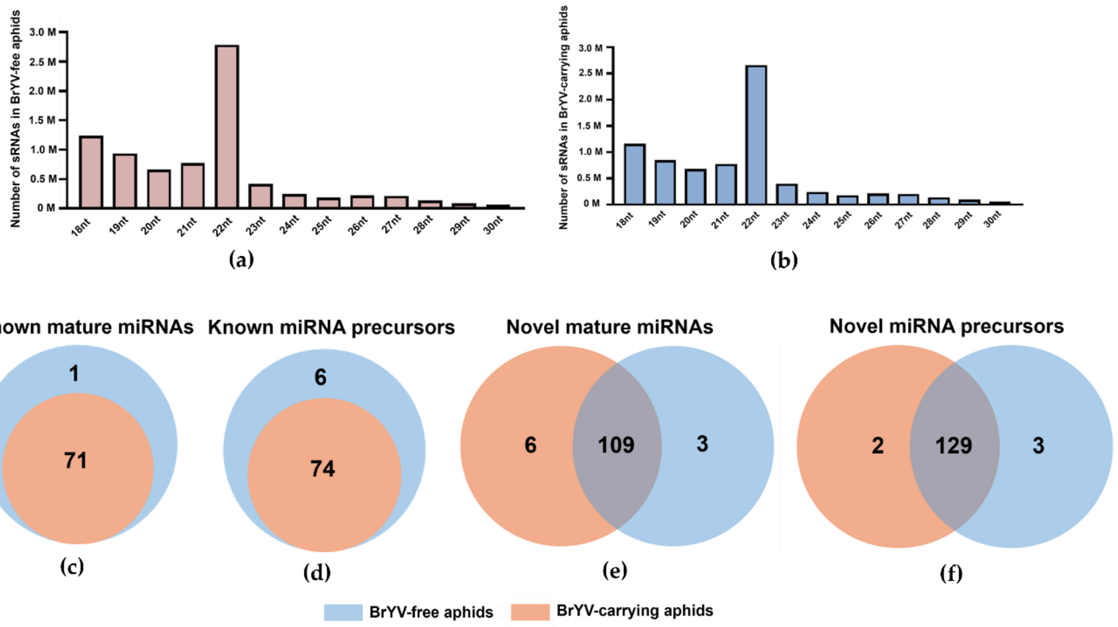

Figure S3. Host-derived sRNAs in BrYV-free and BrYV-carrying aphids under treatment 2. Size distribution of small RNA reads generated in BrYV-free aphids **(a)** and BrYV-carrying aphids **(b)**. **(a, b)** The X-axis represents the length of sRNAs and "M" means million. Venn diagram indicated the number of known **(c, d)** and novel **(e, f)** mature miRNAs **(c, e)** or precursors **(d, f)** that were specific or common to non-viruliferous and viruliferous aphids .

Table S1. Primers used for BrYV detection and RT-qPCR validation.

Gene name	Sequence	Amplicon size (bp)
actin	F: CGGTTCAAAAACCCAAACCAG<br>R: TGGTGATGATTCCGTGTTC	260
BrC2674	F: GATTGTTCTGGTTTTGACTGG	1126
BrC3778	R: TTATACTCATGGTAGGCCTTGAG	
qPCR-actin	F: GGTGTCTCACACACAGTGCC	142
qPCR-actin	R: CGGCGGTGGTGGTGAAGCTG	
novel_7	F: CGAAGGTAACTAGATCACTATGGGA	
mpe-miR-10	F: ACCCTGTAGATCCGAATTTGT	
mpe-miR-276	F: TAGGAACTTCATACCGTGCTCT	
mpe-miR-137	F: CGTATTGCTTGAGAATACACGTAG	
mpe-miR-100	F: GACCCGTAGATCCGAACTTGTG	
novel_199	F: GCGTCCACTGTAGAATTTTGC	
novel_65	F: GCAAGGTAACTTTTTCAATGTGT	
novel_44	F: TGTGATGTGCATGTGGGCTTCT	
novel_136	F: AAACGATTGATTGTTTGACCTATT	

Table S2. Summary of small RNA sequencing results under treatment 2.

Items	Non-viruliferous Aphids	Viruliferous Aphids
Raw reads number	16,670,135	16,932,611
Q30 of raw reads（%）	92.10	94.85
GC content（%）	48.63	49.46
Clean reads number	16,191,644	16,481,704
Total reads[a]	7,688,479	7,135,038

[a] The number of clean reads in length of 18-nt to 25-nt.

Table S3. The known mature miRNAs identified in our data and *M. persicae nicotianae.*

Common miRNAs	Unique miRNAs in our data	Unique miRNAs in *M. persicae nicotianae.*
mpe-bantam, mpe-let-7, mpe-miR-1<br>mpe-miR-10, mpe-miR-100<br>mpe-miR-1000, mpe-miR-124<br>mpe-miR-137, mpe-miR-13a<br>mpe-miR-14, mpe-miR-184a<br>mpe-miR-184b, mpe-miR-190<br>mpe-miR-210, mpe-miR-252a<br>mpe-miR-252b, mpe-miR-263a<br>mpe-miR-263b, mpe-miR-275<br>mpe-miR-276, mpe-miR-2765<br>mpe-miR-277, mpe-miR-278<br>mpe-miR-2796, mpe-miR-279a<br>mpe-miR-279b, mpe-miR-281<br>mpe-miR-29, mpe-miR-2a<br>mpe-miR-2b, mpe-miR-2c<br>mpe-miR-3016, mpe-miR-3024<br>mpe-miR-3027, mpe-miR-3031<br>mpe-miR-3032, mpe-miR-3037<br>mpe-miR-3038, mpe-miR-3039<br>mpe-miR-3040, mpe-miR-3041<br>mpe-miR-3049, mpe-miR-3050<br>mpe-miR-3051, mpe-miR-306<br>mpe-miR-315,mpe-miR-317<br>mpe-miR-34, mpe-miR-7<br>mpe-miR-71, mpe-miR-8<br>mpe-miR-87a, mpe-miR-87b<br>mpe-miR-927, mpe-miR-929<br>mpe-miR-92a, mpe-miR-92b<br>mpe-miR-965, mpe-miR-993<br>mpe-miR-996, mpe-miR-998<br>mpe-miR-9a, mpe-miR-9b<br>mpe-miR-iab-4	mpe-miR-219<br>mpe-miR-3029<br>mpe-miR-3042<br>mpe-miR-3047<br>mpe-miR-307<br>mpe-miR-316<br>mpe-miR-971<br>mpe-miR-981	mpe-miR-3030<br>mpe-miR-3053
